# R-DOTAP Cationic Lipid Nanoparticles Outperform Squalene-Based Adjuvant Systems in Elicitation of CD4 T Cells after Recombinant Influenza Hemagglutinin Vaccination

**DOI:** 10.3390/v15020538

**Published:** 2023-02-15

**Authors:** Thomas R. Henson, Katherine A. Richards, Siva K. Gandhapudi, Jerold G. Woodward, Andrea J. Sant

**Affiliations:** 1 David H. Smith Center for Vaccine Biology and Immunology, Department of Microbiology and Immunology, University of Rochester, Rochester, NY 14642, USA; 2 Department of Microbiology, Immunology and Molecular Genetics, University of Kentucky School of Medicine, Lexington, KY 40506, USA

**Keywords:** adjuvants, CD4 T cells, vaccination, influenza, hemagglutinin

## Abstract

It is clear that new approaches are needed to promote broadly protective immunity to viral pathogens, particularly those that are prone to mutation and escape from antibody-mediated immunity. Prototypic pathogens of this type are influenza and SARS-CoV-2, where the receptor-binding protein exhibits extremely high variability in its receptor-binding regions. T cells, known to target many viral proteins, and within these, highly conserved peptide epitopes, can contribute greatly to protective immunity through multiple mechanisms but are often poorly recruited by current vaccine strategies. Here, we have studied a promising novel pure enantio-specific cationic lipid 1,2-dioleoyl-3-trimethylammonium-propane (R-DOTAP), which was previously recognized for its ability to generate anti-tumor immunity through the induction of potent cytotoxic CD8 T cells. Using a preclinical mouse model, we have assessed an R-DOTAP nanoparticle adjuvant system for its ability to promote CD4 T cell responses to vaccination with recombinant influenza protein. Our studies revealed that R-DOTAP consistently outperformed a squalene-based adjuvant emulsion, even when it was introduced with a potent TLR agonist CpG, in the ability to elicit peptide epitope-specific CD4 T cells when quantified by IFN-γ and IL-2 ELISpot assays. Clinical testing of R-DOTAP containing vaccines in earlier work by others has demonstrated an acceptable safety profile. Hence, R-DOTAP can offer exciting opportunities as an immune stimulant for next-generation prophylactic recombinant protein-based vaccines.

## 1. Introduction

There is an urgent need to develop more effective vaccine approaches to induce broadly protective responses to influenza [1,2,3,4]. The ability of the hemagglutinin (HA) receptor of influenza to drift and mutate to escape immune pressure has prompted efforts to re-target the focus of the B cell response to vaccination. There have been numerous strategies that have been implemented that seek to redirect the B cell response toward the genetically conserved HA stalk domain [5,6,7,8]. These strategies include vaccine constructs composed exclusively of the stalk domain ([9] and reviewed in [10]) or vaccination using chimeric HA constructs with “exotic” rare head domains and conserved stalk domains designed to focus the elicited B cell repertoire on the stalk region of circulating HA [11,12] proteins. There are also molecular strategies that seek to induce HA head-specific antibodies that are expected to protect against a variety of influenza viruses expressing diverse HA antigens [13,14,15,16]. Finally, there are research groups that advocate targeting less genetically variable influenza targets, relative to HA (reviewed in [17]) including NA (reviewed in [18,19,20,21]), NP [22,23,24,25], or M2 [26,27,28].

The complementary approach that we, and others, advocate involves implementing vaccination strategies that elicit CD4 T cells that can provide broadly protective effector functions [29,30,31,32,33,34]. It is now well accepted that CD4 T cells can provide a multiplicity of functions that contribute to protective immunity to influenza. One of the most highly documented functions of CD4 T cells is their ability to potentiate high affinity class switched antibody responses to HA, which is a function conveyed by follicular helper cells (Tfh) (reviewed in [35,36,37]). Beyond the provision of ‘help’ to B cells, CD4 T cells can also promote CD8 T cell expansion and memory through the provision of IL-2 [38] and can produce anti-viral cytokines such as IFN-γ [39]. Finally, subsets of CD4 T cells convey cytotoxic activity [40,41,42] which include many similarities to CD8 T cells, although they are lower in abundance. An important advantage of CD4 T cells is their broad epitope specificity, targeting most viral proteins and within those proteins, reacting to many different peptide epitopes [43]. These features allow CD4 T cells to, in general, be less susceptible to loss in reactivity to variant viruses. The potential for cross-reactive CD4 T cells has been found in many studies, including those that investigated CD4 T cell immunity when a novel pandemic H1N1 influenza strain emerged in 2009 [44,45]. When the persistence of CD4 and CD8 T cell reactivity in humans was tested, cross-reactivity was also found, which was likely due to the exceptionally broad epitope coverage and sequence conservation, even for receptor binding proteins such as influenza HA or SARS-Co-V-2 spike proteins. For both viruses, much of the CD4 T cell reactivity was maintained for HA [46,47] or SARS-CoV-2 spike [48,49] as well as more genetically conserved internal virion proteins such as influenza nucleoprotein and SARS-nucleocapsid (reviewed in [43,50,51,52]).

The broad epitope specificity and diverse protective effector functions of CD4 T cells make it imperative to investigate how best to potentiate their recruitment after vaccination. Although one can detect CD4 T cell responses to traditional inactivated virus vaccines or recombinant protein vaccines, the response is often modest. Adjuvants are routinely used to enhance the immunogenicity of recombinant protein-based vaccines. Although protein-based influenza vaccines can generate immune responses without the addition of adjuvants, adjuvanted formulations generally induce stronger responses in immunocompetent individuals and can rescue functional responses in elderly or immune compromised populations. Furthermore, adjuvants provide significant dose sparing and generate T cell immunity. Current adjuvanted flu vaccines in the market contain squalene-based adjuvants such as MF59.

In this study, we have analyzed the impact of adjuvants in promoting the recruitment of CD4 T cells after recombinant protein vaccination. We were intrigued by a newly developed novel adjuvant called R-DOTAP, which is an enantiospecific cationic lipid nanoparticle that has shown great promise as an antigen delivery system and that has shown safety in Phase I and Phase II clinical trials (NCT02065973, NCT04260126, NCT04580771, NCT05232851) [53]. In animal models, R-DOTAP has primarily been studied for CD8 T cell responses and tumor-specific immunity [54,55,56,57]. We hypothesized that many of the properties of R-DOTAP that resulted in robust CD8 T cell effector differentiation would also result in effective CD4 T cell differentiation in response to recombinant protein antigens. These include facilitated protein uptake to antigen-presenting cells, dendritic cell activation and induction of chemokine expression [55,58]. Recent studies indicate that the R-enantiomer of DOTAP is the primary active component of the racemic mixture of R-and S-DOTAP [59], and it induces Type I IFN responses in the anti-tumor response that are dependent on MyD88 and endosomal TLR-7 and TLR-9 [56]. These features collectively suggest that R-DOTAP is a promising candidate for inducing CD4 T cell cellular immunity that could promote protective immunity to pathogens such as influenza. Here, we analyzed the potency of R-DOTAP in inducing epitope-specific cytokine producing CD4 T cells to a recombinant influenza antigen, using traditional squalene-based adjuvants as comparators. Using a mouse model of vaccination, we found striking enhancement in the recruitment of antigen-specific CD4 T cells in the primary response in R-DOTAP vaccinated mice relative to control adjuvants.

## 2. Materials and Methods

### 2.1. Mice and Ethics Statement

C57BL/6 mice were purchased from NCI. All mice were used between 2 and 5 months of age and were maintained in a specific-pathogen-free facility at the University of Rochester Medical Center according to institutional guidelines. All animal protocols adhere to the AAALAC International, the Animal Welfare Act and the PHS Guide, and they were approved by the University of Rochester Committee on Animal Resources, Animal Welfare Assurance Number A3291-01. The protocol under which these studies were conducted was originally approved on 4 March 2006 (protocol no. 2006-030) and has been reviewed and re-approved every 36 months with the most recent review and approval 29 December 2020.

### 2.2. Preparation of R-DOTAP Nanoparticles and Vaccine Formulations

Current good manufacturing practice grade (CGMP) R-DOTAP was provided by PDS Biotechnology Corporation, Florham Park, NJ, USA. For making vaccine formulations, concentrated antigens dissolved in PBS buffer were diluted to the desired concentration in 280 mM sucrose. Prior to vaccination, the vaccine components were brought to ambient temperature, and the protein antigen component was then mixed at a 1:1 ratio with the R-DOTAP nanoparticles using a pipette to form a uniform suspension. For vaccination, a 100 μL volume was used for each dose, delivered at two sites, either subcutaneously in the rear footpads or intramuscular in the thigh.

### 2.3. Proteins and Peptides

The 15-mer peptides from HA were obtained from Mimotopes. The sequences are listed in Table 1 [60]. Hemagglutinin HA recombinant protein made in baculovirus from influenza virus B/Malaysia/2506/2004 (NR-15172) was obtained from BEIR.

### 2.4. Protein Immunizations

C57BL/6 mice were immunized with 5 μg of B/Malaysia/2506/2004 HA protein emulsified in adjuvant using a 1:1 ratio of adjuvant and antigen diluted in appropriate buffer. For R-DOTAP, antigen was diluted in 280 mM sucrose. For AddaVax (Invivogen San Diego, CA, USA), antigen was diluted in PBS with CpG (2.5 μg per mouse) (ODN1826, IDT, Newark NJ, USA). Ten or eleven days post-vaccination, mice were euthanized, the draining popliteal lymph node (pLN) and spleen were excised and processed into single cell suspensions, red blood cells were lysed from the spleen using ACK lysis buffer and CD4 T cells were isolated by MACS (Miltenyi Biotec, Gaithersburg, MD, USA) negative selection following the manufacturer’s recommendations. Purified CD4 T cells were subsequently used in ELISpot assays, as described below.

### 2.5. ELISpot Assays

ELISpot assays to detect cytokine-producing cells were performed as previously described [60]. Briefly, 96-well filter plates were coated with 2 μg/mL purified rat anti-mouse IL-2 or IFN-γ (clone JES6-1A12 and clone AN-18, respectively, BD Biosciences, San Jose, CA, USA) in PBS overnight at 4 °C, washed with media (complete DMEM media with 10% FBS (Gibco, Grand Island, NY, USA)) to remove any unbound antibody and incubated with 100 μL media per well for 1 hr to block non-specific protein binding in subsequent steps. Isolated CD4 T cells, plated at the optimal concentration to enumerate cytokine-producing cells (100,000–250,000 for pLN and 300,000 for splenocytes) were co-cultured with 500,000 syngeneic spleen cells, which were used as the source of antigen-presenting cells (APC) and peptide, at a final concentration of 5 μM in media in a total final volume of 200 μL for 18–20 h at 37 °C and 5% CO_2_. The cells were removed from the plates, and the plates were washed with wash buffer (1X PBS, 0.1% Tween-20). Biotinylated rat anti-mouse IL-2 or IFN-γ (clone JES6-5H4 and clone XMG1.2, respectively, BD Biosciences) was added, at a concentration of 2 μg/mL (50 μL/well) in wash buffer with 10% FBS and incubated at room temperature for 30 min. The plates were washed again and streptavidin-conjugated alkaline phosphatase (Jackson ImmunoResearch, West Grove, PA, USA) was added at a dilution of 1:1000 in wash buffer with 10% FBS, 50 μL/well, and incubated for 30 min at room temperature. The plates were washed with wash buffer and developed using Vector Blue substrate kit III (Vector Laboratories, Newark, CA, USA) prepared in 100 mM Tris, pH 8.2. After drying, the quantification of spots was performed with an Immunospot reader series 5.2, using Immunospot software, version 5.1. All culture conditions were replicated in duplicate wells. Control wells containing APC, CD4 T cells and media with no added peptide were used for negative control, background responses. Background responses ranged from 0 to 16 spots per million for IFN-γ and 6 to 60 spots per million for IL-2. Generally, the higher background responses were exhibited in the spleen-derived samples. Representative ELISpot plate images are shown in Figures S1 and S2 for IL-2 and IFN-γ, respectively. Results are presented as the mean ± standard error of the mean (SEM) or range of the response with background subtracted. Statistical analyses were performed using GraphPad software version 9.1.

### 2.6. ELISA Assays

Serum was collected at D11 and D31 post vaccination. HA-specific antibodies in the sera from individual mice were quantified by ELISA assays. Plates (Corning Costar, Tewksbury, MA, USA) were coated with 200 ng of purified HA protein from influenza B/Brisbane/60/08. Wells were rinsed with PBS, incubated with blocking buffer (3% BSA in PBS), and then diluted serum samples (in 0.5% BSA–PBS) were added to the plates and incubated for 2 h at room temperature. The wells were washed and incubated sequentially with 100 μL/well alkaline phosphatase conjugated goat anti-mouse IgG secondary antibody (SouthernBiotech, Birmingham, AL, USA) and one *p*-nitrophenyl phosphate substrate (Sigma, Burlington, MA, USA). After washing, absorbance at 405 nm was read.

## 3. Results

### 3.1. HA-B as a Model Antigen for Broadly Distributed CD4 T Cell Epitopes

**Figure 1:**
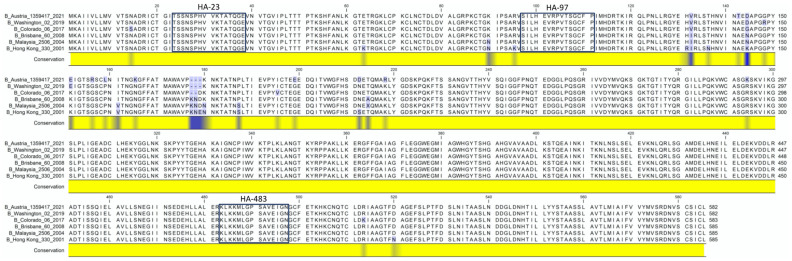
Influenza B HA sequences from the Victoria lineage that have been included in seasonal influenza vaccines over the past 20 years have been aligned to indicate sequence conservation. Yellow indicates conserved regions and blue indicates divergent amino acids. Indicated in the boxes are the three dominant CD4 T cells peptide epitopes. The sequence accession numbers are: B/Washington/02/2019 QCG86180, B/Colorado/06/2017 ARQ85589, B/Brisbane/60/2008 ANC28539, B/Malaysia/2506/2004ACR15732 and B/Hong Kong/330/2001 ABL77178.

In these studies, the influenza HA-B protein (B/Malaysia/2506/2004) was used as a prototype for recombinant HA proteins. Recombinant antigens are increasingly used as the basis for vaccines; the most notable is FluBlok, which is a licensed recombinant quadrivalent HA protein influenza vaccine (reviewed in [61,62]). We used the C57BL/6 (B6) mouse model for these studies in order to enable future genetic manipulations in the host that would help identify key genes/proteins that were involved in the CD4 T cell response phenotype or magnitude. Many genetically modified strains are uniquely available in the B6 background [63]. Previous studies by our group identified three major CD4 T cell epitopes in HA-B following the influenza infection of B6 mice [60], offering a good opportunity to broadly assess CD4 T cell specificity and immunodominance. Figure 1 shows the location of these epitopes in the HA-B protein as well as their degree of conservation across HA-B proteins from historical and contemporary strains of influenza B.

### 3.2. Evaluation of Three Adjuvant Systems Reveals the Exceptional Potency of R-DOTAP in Elicitation of CD4 T Cells

**Figure 2:**
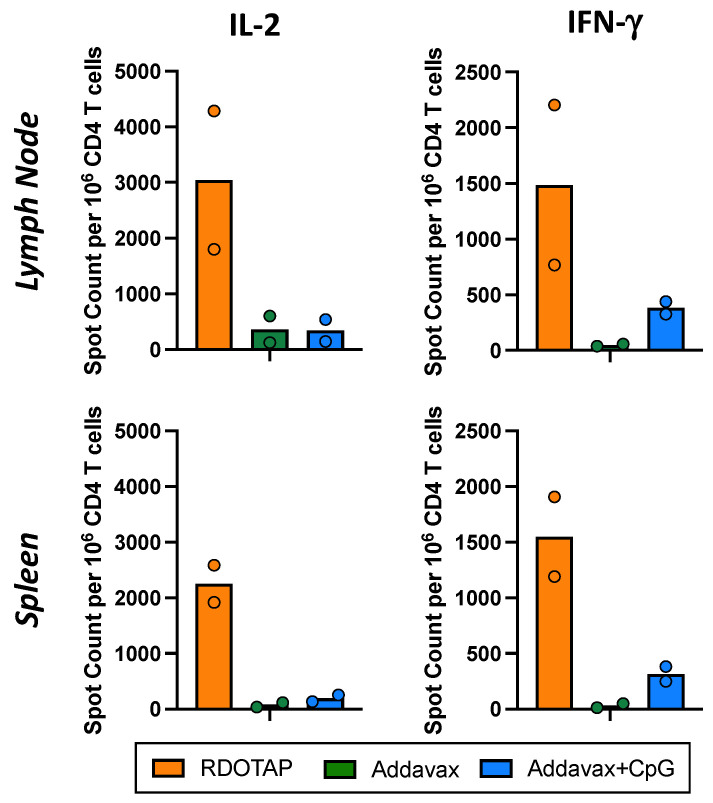
CD4 T cell response to influenza B HA following subcutaneous protein vaccination in three adjuvant systems, R-DOTAP (orange), AddaVax (green) and AddaVax + CpG (blue). CD4 T cells were isolated from the draining lymph nodes and spleen 10–11 days post vaccination and assayed for cytokine production using IL-2 (**left**) and IFN-γ (**right**) ELISpot assays. The frequency of cytokine-producing CD4 T cells in the draining lymph node (**top**) and spleen (**bottom**) in response to the HA-B peptides (summed) is shown. Background (APC + T cells with media) was subtracted from all responses. This is the average of two independent experiments with the individual experiments indicated as circles. Overall, 3–4 mice were pooled in each experiment.

The first experiments explored the ability of R-DOTAP to elicit peptide epitope-specific CD4 T cells after primary vaccination, relative to the commonly used squalene-based adjuvant AddaVax, which is analogous to the human adjuvant MF59 [64,65] and is commercially available from InvivoGen for animal studies (see Section 2). AddaVax was tested alone or in combination with the TLR9 agonist CpG [66] and compared to R-DOTAP, using HA-B as the vaccine antigen. Mice were vaccinated subcutaneously in the hind footpad, and draining popliteal lymph nodes (LN) were used as a source of CD4 T cells at day 11 post-vaccination. CD4 T cell populations were purified from single cell suspensions isolated from the draining LN or spleen by the depletion of other subsets of cells, including B cells and CD8 T cells. CD4 T cells were analyzed for reactivity to HA-B using peptide-stimulated cytokine ELISpot assays (Figure 2). Cells isolated from the draining lymph node (top) or spleen (bottom) were assayed for the production of IL-2 (left) or IFN-γ (right). Both of these cytokines are critical for protective immunity. IL-2 facilitates T cell expansion and CD8 T cell memory and IFN-γ has a multiplicity of functions, including direct antiviral activity in the respiratory tract, upregulation of MHC proteins on antigen-presenting cells and other cells in the respiratory tract and IgG isotype switch. Figure 2 shows the sums of the responses to the three peptides, allowing a generalized view of the immunogenicity of HA-B when introduced with R-DOTAP (orange bars), AddaVax alone (green bars), or AddaVax with added CpG at 2.5μg per mouse. The results from two independent experiments with the range in responses are shown and are presented as cytokine-producing cells per million CD4 T cells with background subtracted. For both IL-2 and IFN-γ, R-DOTAP-elicited responses exhibited a substantially more robust response to HA-B than either AddaVax alone or AddaVax with added CpG. IL-2-producing cells in the R-DOTAP-adjuvanted vaccine averaged approximately 2000–3000 IL-2-producing CD4 T cells, depending on the tissue sampled and approximately 1500 IFN-γ-producing cells per million. In contrast, AddaVax-adjuvanted HA-B elicited less than 500 spots per million of IL-2-producing cells and almost undetectable levels of IFN-γ-producing cells. The addition of CpG to the AddaVax led to detectable gains in the elicitation of IFN-γ-producing cells but only to a level that was approximately one-third of that elicited by R-DOTAP. This impact of CpG is consistent with the view of this component as a TLR-9 agonist, primarily enhancing the development of a Th1 response.

**Figure 3:**
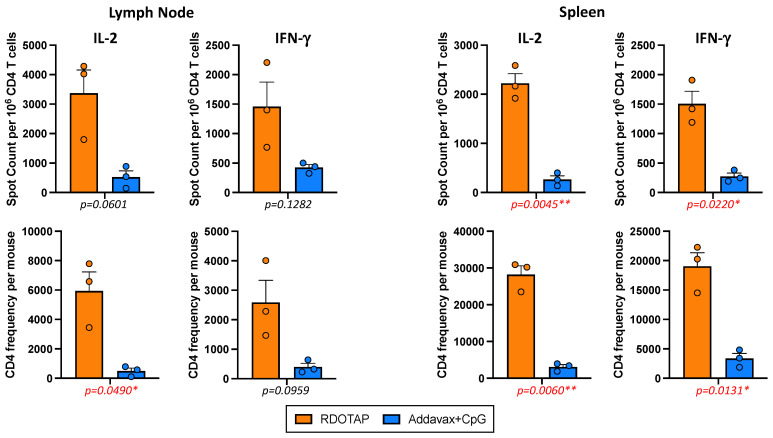
Mice were immunized subcutaneously with B/Malaysia HA protein emulsified in the indicated adjuvant (R-DOTAP, orange or AddaVax + CpG, blue), and tissues were harvested at day 10/11 post vaccination. Tissues from 3–4 mice were pooled in each experiment. The HA-B reactive CD4 T cells in the draining popliteal lymph node (**left**) and the number of HA-B reactive CD4 T cells in the spleen (**right**) detected by IL-2 and IFN-γ ELISpot. The top graphs show the frequency of HA-B epitope specific cytokine-producing CD4 cells per million and the bottom graphs illustrate the HAB epitope-specific cytokine-producing cells per mouse. All data have been corrected for background. Shown as a bar is the average of three independent experiments with each experiment shown as a circle and the standard error of the mean indicated. Below the plot is the *p*-value calculated using an unpaired *t*-test with Welch’s correction (* *p* ≤ 0.05 and ** *p* ≤ 0.01).

Because of the desired multifunctionality of vaccine-elicited CD4 T cells, we chose to compare only R-DOTAP and AddaVax + CpG for the remainder of the experiments, exploring antigen specificity and different modes of vaccination. Figure 3 shows the results of three additional and independent experiments that illustrate the quantitative advantages of R-DOTAP (in orange), relative to AddaVax/CpG (in blue), with statistical values shown below each panel. The responses of cells isolated from the lymph node are shown on the left and the spleen responses are shown on the right. In these experiments, the frequency of reactive CD4 T cells per million CD4 T cells is shown in the top panels and the total peptide-specific CD4 T cells per mouse is shown on the bottom. The secondary calculation, combining the frequency of peptide-reactive cells and total yield of CD4 T cells isolated from the draining lymph node or spleen, allows us to factor in the overall differences in yields of CD4 T cells elicited by the two adjuvant systems, which is approximately 2–3 fold higher with R-DOTAP in the primary draining popliteal lymph node (see Table 2). The average “fold-difference” in the total HA-B specific CD4 T cells recruited in the response to AddaVax/CpG vs. R-DOTAP ranged from 9-fold to 12-fold for IL-2 and 5.6 to 6.5-fold for IFN-γ (Table 3), illustrating the greater overall efficacy of the R-DOTAP adjuvant. Most of the differences approached or achieved statistically significant values, as indicated below each panel.

### 3.3. Vaccination with Recombinant HA-B Protein Elicits a Balanced Epitope Distribution

**Figure 4:**
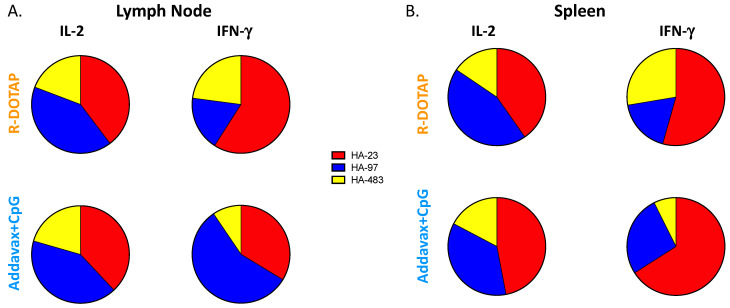
Mice were immunized subcutaneously with B/Malaysia HA protein in adjuvant and tissues were harvested at day 10/11 post-vaccination. Shown are pie diagrams of the average response from three independent experiments illustrating the fraction of the response dedicated to each of the peptide epitopes, with R-DOTAP on the top and AddaVax + CpG on the bottom. To the left (**A**) is the response in the draining popliteal lymph node and to the right (**B**) is the response in the spleen detected by IL-2 and IFN-y ELISpot. There was no statistical difference in the relative immunodominance between R-DOTAP and AddaVax + CpG.

With the goal of preclinical studies of adjuvants to explore the potential to elicit CD4 T cells that can recognize conserved epitopes distributed across vaccine antigens, we evaluated the distribution of peptide epitope specificities detectable in the CD4 T cells elicited by the adjuvant systems tested. Figure 4 shows the epitope distribution and immunodominance hierarchy induced by the two HA-B adjuvant combinations, where the relative frequency of the major epitopes relative to the total response is shown by different colors in pie diagrams. These peptide epitopes are denoted here as HA-23, HA-97 and HA-483 (see Table 1), where the nomenclature is based on the first amino acid number of the 17-mer peptide identified through epitope discovery using the strategy of overlapping peptide libraries [67,68,69,70]. These results show that when IL-2-producing cells were quantified, both adjuvants elicited a balanced response across the 3 HA-B epitopes, although the abundance of CD4 T cells was significantly greater with R-DOTAP as an adjuvant. IFN-γ-producing cells tended to be enriched for specificity toward HA-23 with both adjuvants. These differences, across the three independent experiments, were not statistically significant.

### 3.4. Intramuscular Vaccination with R-DOTAP-HA-B Reveals Dramatic Outperformance of R-DOTAP, Relative to AddaVax-CpG

**Figure 5:**
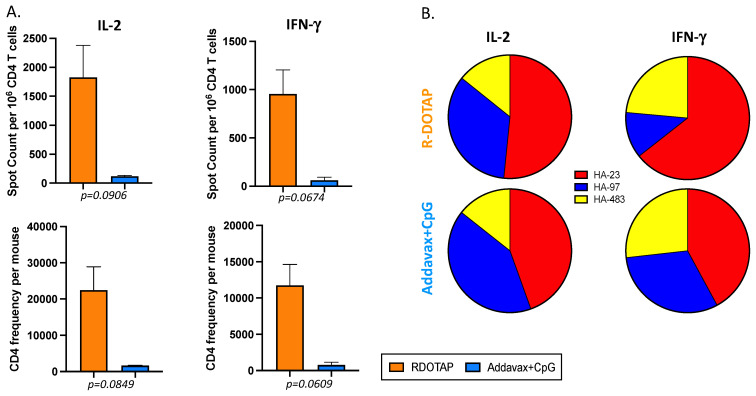
CD4 T cell response following intramuscular vaccination with HA-B. Mice (3–4 per group per experiment) were immunized intramuscularly with HA B/Malaysia in adjuvant and spleens were harvested at day 10/11 post vaccination and used as a source of CD4 T cells, enriched by depletion of other subsets of cells. Panel (**A**). The magnitude of the response in the spleen detected by IL-2 and IFN-γ ELISpot. The top plots show the frequency of cytokine producing CD4 cells per million and the bottom groups illustrate the cytokine producing cells calculated per mouse. All data have been corrected for background. Shown is the average of three independent experiments and the standard error of the mean. Below each plot is the *p*-value calculated using an unpaired t-test with Welch’s correction. Panel (**B**). Pie diagrams illustrating the fraction of the response dedicated to each of the HA peptide epitopes that were tested, with R-DOTAP on the top and Addavax + CpG on the bottom. The cytokine detected is indicated above. Shown is the average of three independent experiments. There was no statistical difference detected between the adjuvant systems.

The previous experiments involved subcutaneous vaccination. Humans are most often vaccinated with inactivated influenza vaccines via intramuscular injection. To examine the immunogenicity of recombinant HA-B induced by the presence of the two alternative adjuvants following IM injection, B6 mice were vaccinated with the HA-B adjuvant combination, and the responses of splenic CD4 T cells were than assayed for specificity and functionality using peptide-stimulated cytokine ELISpot assays. Figure 5 shows the results of these experiments, in Figure 5A, showing both the frequency of IL-2 (left panels) and IFN-γ (right)-producing, HA-B epitope-specific cells. The top panels show the frequency of reactive cells per million CD4 T cells and the bottom panels show the total number of epitope-specific cells recovered from the spleen. The average difference in both frequency and total numbers ranged from 13.5 to 15-fold. The differences approached, but did not reach, statistical significance, with the *p* values shown beneath each value. The peptide-specific epitope distribution is shown in Figure 5B, showing a balanced average epitope distribution across the three conserved epitopes that did not differ significantly for the two adjuvant systems. Thus, the overall benefit of R-DOTAP vs. AddaVax/CpG in eliciting CD4 T cells was replicated using intramuscular injection. Overall, these data suggest that R-DOTAP, which has the potential to be used in human subjects, offers dramatic advantages to the MF59-like adjuvant AddaVax, even when AddaVax is supplemented with the robust TLR-9 agonist, CpG.

### 3.5. Antibody Production

**Figure 6:**
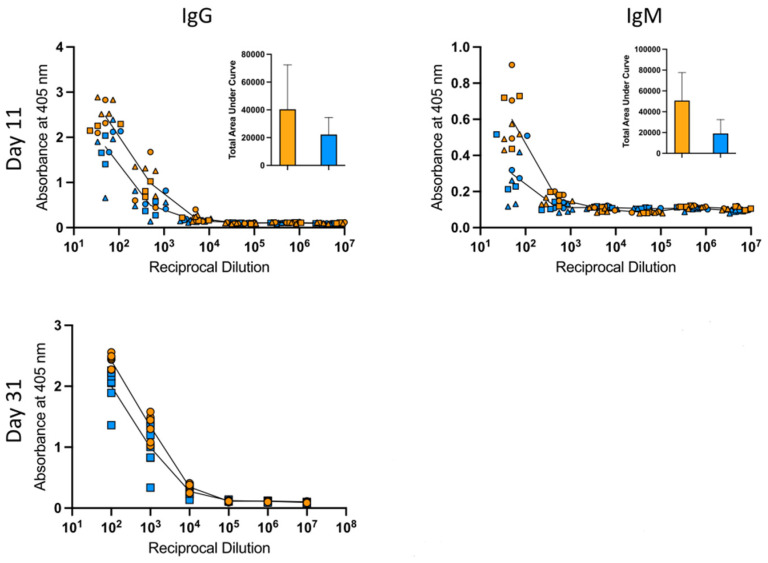
HA-B specific serum IgG and IgM responses. Mice were immunized subcutaneously with B/Malaysia HA protein in adjuvant, and serum was collected at days 11 and day 31 post-immunization. Serum samples from R-DOTAP (orange) and AddaVax + CpG (blue) were tested for IgG and IgM antibodies specific to recombinant HA B/Brisbane/60/08 by ELISA assay. Show are the dilution curves and each individual mouse (10 mice at D11 and 7 mice at D31). Shown in the inset are the total area under the curve calculations at D11. No differences between the R-DOTAP and AddaVax + CpG groups reached statistical significance.

The elicitation of antibodies is a key parameter by which most vaccines are evaluated. To examine the ability of R-DOTAP to elicit HA-B specific antibodies, ELISA assays were performed using serum collected at day 11 (the peak of the primary response) and at day 31. Figure 6 shows the results of these experiments on individual mice, quantifying total IgG and IgM. Both adjuvant systems elicited readily detectable IgG and IgM antibodies after a single vaccination and IgG levels were enhanced, as expected, at the late time point tested. Similar patterns were observed for individual IgG isotypes (data not shown). Although there was a modest trend toward a greater production of antibody in the R-DOTAP/HA-B-vaccinated mice, these serum antibody levels were not statistically different between the two adjuvant systems.

## 4. Summary and Discussion

The studies presented here indicate that the cationic lipid nanoparticle R-DOTAP dramatically enhances CD4 T cell responses to recombinant HA-B proteins, relative to conventional squalene-based emulsion AddaVax, even when the latter adjuvant is potentiated with the strong Th1-inducing TLR9 agonist CpG. The elicited CD4 T cells from R-DOTAP-HA-B vaccinated mice displayed both abundant IFN-γ and IL-2 production, providing multifunctional potentiality that can help both developing CD8 T cell responses (through the production of IL-2) as well as direct anti-viral effects and the potentiation of antigen-presenting function (through the production of IFN-γ). The method used here to measure cytokine production (ELISpot assays) does not allow estimation of the multifunctionality of individual cells: only the population of the elicited CD4 T cells as a whole. Ongoing experiments are evaluating the multifuctionality of individual cells by intracellular cytokine production and flow cytometry. Current experiments are also assessing the production of IL-4 and IL-5, which as Th2 cytokines can also contribute to potent B cell and antibody responses. Preliminary results indicate that Th2 cytokine-producing cells are also robustly elicited by R-DOTAP.

The mechanisms that underlie the greater efficacy of R-DOTAP in the elicitation of antigen-specific CD4 T cells is likely due to at least three factors. First, the cationic charge on R-DOTAP promotes efficient binding of the lipid nanoparticle and its cargo to antigen-presenting cells such as dendritic cells [56]. In addition, likely central to the activity of R-DOTAP as an antigen delivery system is the induction of TLR-7 and TLR-9 signaling and production of type I IFN [56] at the site in vivo where the immune response is initiated. The prototype IFN-γ- and IL-2-producing CD4 T cells noted in our studies here are similar to what we have observed in animal models of influenza infection [60,71]. Finally, the efficacy of R-DOTAP in the induction of Type I IFN has been shown to upregulate CD69 expression in innate cells and T cells in the vaccine draining lymph node [60], perhaps offering the opportunity for the antigen-specific T cells to repeatedly engage antigen-presenting cells prior to leaving the lymph node, which may promote expansion and effector differentiation.

Finally, although some studies [72,73,74] have shown that the activity of R-DOTAP can be enhanced by addition of TLR ligands, here, we show that R-DOTAP alone can promote the activation, expansion and differentiation of CD4 T cells comprised of Th1 phenotype, offering a simpler and safer path for use in human vaccination efforts. The combined protective effects of antigen-specific CD8 T cells and CD4 T cells by this adjuvant system, when administered for pathogen-specific immunity, thus has the potential to provide a comprehensive broadly protective state in the host.

## Figures and Tables

**Table 1:** Influenza B B/Brisbane/60/2008 HA peptide sequences.

Peptide Name	Amino Acids	Sequence
HA-23	23-38	TSSNSPHVVKTATQGE
HA-97	97-111	SILHEVRPVTSGCFP
HA-483	483-497	KLKKMLGPSAVEIGN

**Table 2:** CD4 T cell yields following purification from subcutaneous vaccination with HA B/Malaysia protein emulsified in R-DOTAP or AddaVax + CpG.

	Adjuvant	Exp1	Exp2	Exp3	Average
**pLN**	R-DOTAP	4 × 10^6^	5.4 × 10^6^	7.6 × 10^6^	6.0 × 10^6^
	Addavax + CpG	2 × 10^6^	2.1 × 10^6^	5.8 × 10^6^	3.3 × 10^6^
**Spleen**	R-DOTAP	2.1 × 10^7^	1.4 × 10^7^	1.2 × 10^7^	1.6 × 10^7^
	Addavax + CpG	1.1 × 10^7^	1.7 × 10^7^	1.4 × 10^7^	1.4 × 10^7^
**Number of Mice**		3	3	4	

**Table 3:** Following subcutaneous vaccination with HA B/Malaysia in adjuvant and cytokine ELISpot using CD4 T cells isolated from the draining lymph node and spleen, as shown in Figure 3, the average frequencies of cytokine producing CD4 T cells per million corrected for background, the average number of cytokine-producing CD4 T cells per mouse corrected for background and fold differences between R-DOTAP and AddaVax + CpG were calculated.

		Lymph Node			Spleen		
		R-DOTAP	AddaVax + CpG	Fold Difference	R-DOTAP	AddaVax + CpG	Fold Difference
**Frequency**	IL-2	3370	523	6.4	2223	265	8.4
	IFN-γ	1458	421	3.5	1506	273	5.5
**CD4 T cells per mouse**	IL-2	5934	488	12	28,211	3086	9
	IFN-γ	2586	398	6.5	19,024	3367	5.6

## Data Availability

The full complement of data accumulated for these studies is available upon reasonable request to the corresponding author.

## Supplementary Materials

The following supporting information can be downloaded at: https://www.mdpi.com/article/10.3390/v15020538/s1, Figure S1: Represtenative plate image for IL-2 ELISpot and Figure S2: Representative plate image for IFN-γ ELISpot.

## References

[1] Khalil N., Bernstein D.I. (2022). Influenza vaccines: Where we are, where we are going. Curr. Opin. Pediatr..

[2] Kim H., Webster R.G., Webby R.J. (2018). Influenza Virus: Dealing with a Drifting and Shifting Pathogen. Viral. Immunol..

[3] Yamayoshi S., Kawaoka Y. (2019). Current and future influenza vaccines. Nat. Med..

[4] Nachbagauer R., Palese P. (2020). Is a Universal Influenza Virus Vaccine Possible?. Annu. Rev. Med..

[5] Sun W., Zheng A., Miller R., Krammer F., Palese P. (2019). An Inactivated Influenza Virus Vaccine Approach to Targeting the Conserved Hemagglutinin Stalk and M2e Domains. Vaccines.

[6] Steel J., Lowen A.C., Wang T.T., Yondola M., Gao Q., Haye K., Garcia-Sastre A., Palese P. (2010). Influenza virus vaccine based on the conserved hemagglutinin stalk domain. mBio.

[7] Coughlan L., Palese P. (2018). Overcoming Barriers in the Path to a Universal Influenza Virus Vaccine. Cell Host Microbe.

[8] Neu K.E., Henry Dunand C.J., Wilson P.C. (2016). Heads, stalks and everything else: How can antibodies eradicate influenza as a human disease?. Curr. Opin. Immunol..

[9] Corbett K.S., Moin S.M., Yassine H.M., Cagigi A., Kanekiyo M., Boyoglu-Barnum S., Myers S.I., Tsybovsky Y., Wheatley A.K., Schramm C.A. (2019). Design of Nanoparticulate Group 2 Influenza Virus Hemagglutinin Stem Antigens That Activate Unmutated Ancestor B Cell Receptors of Broadly Neutralizing Antibody Lineages. mBio.

[10] Krammer F. (2015). The Quest for a Universal Flu Vaccine: Headless HA 2.0. Cell Host Microbe.

[11] Choi A., Bouzya B., Cortes Franco K.D., Stadlbauer D., Rajabhathor A., Rouxel R.N., Mainil R., Van der Wielen M., Palese P., Garcia-Sastre A. (2019). Chimeric Hemagglutinin-Based Influenza Virus Vaccines Induce Protective Stalk-Specific Humoral Immunity and Cellular Responses in Mice. Immunohorizons.

[12] Isakova-Sivak I., Korenkov D., Smolonogina T., Kotomina T., Donina S., Matyushenko V., Mezhenskaya D., Krammer F., Rudenko L. (2018). Broadly protective anti-hemagglutinin stalk antibodies induced by live attenuated influenza vaccine expressing chimeric hemagglutinin. Virology.

[13] Sautto G.A., Kirchenbaum G.A., Abreu R.B., Ecker J.W., Pierce S.R., Kleanthous H., Ross T.M. (2020). A Computationally Optimized Broadly Reactive Antigen Subtype-Specific Influenza Vaccine Strategy Elicits Unique Potent Broadly Neutralizing Antibodies against Hemagglutinin. J. Immunol..

[14] Skarlupka A.L., Owino S.O., Suzuki-Williams L.P., Crevar C.J., Carter D.M., Ross T.M. (2019). Computationally optimized broadly reactive vaccine based upon swine H1N1 influenza hemagglutinin sequences protects against both swine and human isolated viruses. Hum. Vaccin. Immunother..

[15] Sautto G.A., Kirchenbaum G.A., Ecker J.W., Bebin-Blackwell A.G., Pierce S.R., Ross T.M. (2018). Elicitation of Broadly Protective Antibodies following Infection with Influenza Viruses Expressing H1N1 Computationally Optimized Broadly Reactive Hemagglutinin Antigens. Immunohorizons.

[16] Crevar C.J., Carter D.M., Lee K.Y., Ross T.M. (2015). Cocktail of H5N1 COBRA HA vaccines elicit protective antibodies against H5N1 viruses from multiple clades. Hum. Vaccin. Immunother..

[17] Nguyen Q.T., Choi Y.K. (2021). Targeting Antigens for Universal Influenza Vaccine Development. Viruses.

[18] Giurgea L.T., Morens D.M., Taubenberger J.K., Memoli M.J. (2020). Influenza Neuraminidase: A Neglected Protein and Its Potential for a Better Influenza Vaccine. Vaccines.

[19] Eichelberger M.C., Wan H. (2015). Influenza neuraminidase as a vaccine antigen. Curr. Top Microbiol. Immunol..

[20] Eichelberger M.C., Morens D.M., Taubenberger J.K. (2018). Neuraminidase as an influenza vaccine antigen: A low hanging fruit, ready for picking to improve vaccine effectiveness. Curr. Opin. Immunol..

[21] Wohlbold T.J., Krammer F. (2014). In the shadow of hemagglutinin: A growing interest in influenza viral neuraminidase and its role as a vaccine antigen. Viruses.

[22] Tan M.P., Tan W.S., Mohamed Alitheen N.B., Yap W.B. (2021). M2e-Based Influenza Vaccines with Nucleoprotein: A Review. Vaccines.

[23] Cookenham T., Lanzer K.G., Gage E., Lorenzo E.C., Carter D., Coler R.N., Baldwin S.L., Haynes L., Reiley W.W., Blackman M.A. (2020). Vaccination of aged mice with adjuvanted recombinant influenza nucleoprotein enhances protective immunity. Vaccine.

[24] Joe P.T., Christopoulou I., van Hoecke L., Schepens B., Ysenbaert T., Heirman C., Thielemans K., Saelens X., Aerts J.L. (2019). Intranodal administration of mRNA encoding nucleoprotein provides cross-strain immunity against influenza in mice. J. Transl. Med..

[25] Nelson S.A., Dileepan T., Rasley A., Jenkins M.K., Fischer N.O., Sant A.J. (2021). Intranasal Nanoparticle Vaccination Elicits a Persistent, Polyfunctional CD4 T Cell Response in the Murine Lung Specific for a Highly Conserved Influenza Virus Antigen That Is Sufficient To Mediate Protection from Influenza Virus Challenge. J. Virol..

[26] Deng L., Cho K.J., Fiers W., Saelens X. (2015). M2e-Based Universal Influenza A Vaccines. Vaccines.

[27] Schepens B., De Vlieger D., Saelens X. (2018). Vaccine options for influenza: Thinking small. Curr. Opin. Immunol..

[28] Mezhenskaya D., Isakova-Sivak I., Rudenko L. (2019). M2e-based universal influenza vaccines: A historical overview and new approaches to development. J. Biomed. Sci..

[29] Clemens E.B., van de Sandt C., Wong S.S., Wakim L.M., Valkenburg S.A. (2018). Harnessing the Power of T Cells: The Promising Hope for a Universal Influenza Vaccine. Vaccines.

[30] Elbahesh H., Saletti G., Gerlach T., Rimmelzwaan G.F. (2019). Broadly protective influenza vaccines: Design and production platforms. Curr. Opin. Virol..

[31] Sant A.J., Richards K.A., Nayak J. (2018). Distinct and complementary roles of CD4 T cells in protective immunity to influenza virus. Curr. Opin. Immunol..

[32] Koutsakos M., Nguyen T.H.O., Kedzierska K. (2019). With a Little Help from T Follicular Helper Friends: Humoral Immunity to Influenza Vaccination. J. Immunol..

[33] Nelson S.A., Sant A.J. (2021). Potentiating Lung Mucosal Immunity Through Intranasal Vaccination. Front. Immunol..

[34] Hassert M., Harty J.T. (2022). Tissue resident memory T cells- A new benchmark for the induction of vaccine-induced mucosal immunity. Front. Immunol..

[35] Vinuesa C.G., Linterman M.A., Yu D., MacLennan I.C. (2016). Follicular Helper T Cells. Annu. Rev. Immunol..

[36] Song W., Craft J. (2019). T follicular helper cell heterogeneity: Time, space, and function. Immunol. Rev..

[37] Juno J.A., Hill D.L. (2022). T follicular helper cells and their impact on humoral responses during pathogen and vaccine challenge. Curr. Opin. Immunol..

[38] Liao W., Lin J.X., Leonard W.J. (2013). Interleukin-2 at the crossroads of effector responses, tolerance, and immunotherapy. Immunity.

[39] Bot A., Bot S., Bona C.A. (1998). Protective role of gamma interferon during the recall response to influenza virus. J. Virol..

[40] Juno J.A., van Bockel D., Kent S.J., Kelleher A.D., Zaunders J.J., Munier C.M. (2017). Cytotoxic CD4 T Cells-Friend or Foe during Viral Infection?. Front. Immunol..

[41] Takeuchi A., Saito T. (2017). CD4 CTL, a Cytotoxic Subset of CD4(+) T Cells, Their Differentiation and Function. Front Immunol.

[42] Preglej T., Ellmeier W. (2022). CD4(+) Cytotoxic T cells-Phenotype, Function and Transcriptional Networks Controlling Their Differentiation Pathways. Immunol. Lett..

[43] Sant A.J., DiPiazza A.T., Nayak J.L., Rattan A., Richards K.A. (2018). CD4 T cells in protection from influenza virus: Viral antigen specificity and functional potential. Immunol. Rev..

[44] Sullivan S.J., Jacobson R.M., Dowdle W.R., Poland G.A. (2010). 2009 H1N1 influenza. Mayo Clin. Proc..

[45] Neumann G., Kawaoka Y. (2011). The first influenza pandemic of the new millennium. Influenza Other Respir. Viruses.

[46] Ge X., Tan V., Bollyky P.L., Standifer N.E., James E.A., Kwok W.W. (2010). Assessment of seasonal influenza A virus-specific CD4 T-cell responses to 2009 pandemic H1N1 swine-origin influenza A virus. J. Virol..

[47] Alam S., Sant A.J. (2011). Infection with seasonal influenza virus elicits CD4 T cells specific for genetically conserved epitopes that can be rapidly mobilized for protective immunity to pandemic H1N1 influenza virus. J. Virol..

[48] Liu J., Chandrashekar A., Sellers D., Barrett J., Jacob-Dolan C., Lifton M., McMahan K., Sciacca M., VanWyk H., Wu C. (2022). Vaccines elicit highly conserved cellular immunity to SARS-CoV-2 Omicron. Nature.

[49] Keeton R., Tincho M.B., Ngomti A., Baguma R., Benede N., Suzuki A., Khan K., Cele S., Bernstein M., Karim F. (2022). T cell responses to SARS-CoV-2 spike cross-recognize Omicron. Nature.

[50] Grifoni A., Sidney J., Vita R., Peters B., Crotty S., Weiskopf D., Sette A. (2021). SARS-CoV-2 human T cell epitopes: Adaptive immune response against COVID-19. Cell Host Microbe.

[51] Egan M.A. (2007). Towards the development of a therapeutic vaccine for the treatment of HIV-1 infection: Are we closer than ever?. Expert Rev. Vaccines.

[52] Kedzierska K., Thomas P.G. (2022). Count on us: T cells in SARS-CoV-2 infection and vaccination. Cell Rep. Med..

[53] Smalley Rumfield C., Pellom S.T., Morillon Ii Y.M., Schlom J., Jochems C. (2020). Immunomodulation to enhance the efficacy of an HPV therapeutic vaccine. J. Immunother. Cancer.

[54] Chen W., Yan W., Huang L. (2008). A simple but effective cancer vaccine consisting of an antigen and a cationic lipid. Cancer Immunol. Immunother..

[55] Yan W., Chen W., Huang L. (2007). Mechanism of adjuvant activity of cationic liposome: Phosphorylation of a MAP kinase, ERK and induction of chemokines. Mol. Immunol..

[56] Gandhapudi S.K., Ward M., Bush J.P.C., Bedu-Addo F., Conn G., Woodward J.G. (2019). Antigen Priming with Enantiospecific Cationic Lipid Nanoparticles Induces Potent Antitumor CTL Responses through Novel Induction of a Type I IFN Response. J. Immunol..

[57] Bei R., Guptill V., Masuelli L., Kashmiri S.V., Muraro R., Frati L., Schlom J., Kantor J. (1998). The use of a cationic liposome formulation (DOTAP) mixed with a recombinant tumor-associated antigen to induce immune responses and protective immunity in mice. J. Immunother..

[58] Riehl M., Harms M., Hanefeld A., Baleeiro R.B., Walden P., Mader K. (2017). Combining R-DOTAP and a particulate antigen delivery platform to trigger dendritic cell activation: Formulation development and in-vitro interaction studies. Int. J. Pharm..

[59] Vasievich E.A., Chen W., Huang L. (2011). Enantiospecific adjuvant activity of cationic lipid DOTAP in cancer vaccine. Cancer Immunol. Immunother..

[60] Rattan A., White C.L., Nelson S., Eismann M., Padilla-Quirarte H., Glover M.A., Dileepan T., Marathe B.M., Govorkova E.A., Webby R.J. (2022). Development of a Mouse Model to Explore CD4 T Cell Specificity, Phenotype, and Recruitment to the Lung after Influenza B Infection. Pathogens.

[61] Cox M.M., Patriarca P.A., Treanor J. (2008). FluBlok, a recombinant hemagglutinin influenza vaccine. Influenza Other Respir. Viruses.

[62] Dunkle L.M., Izikson R. (2016). Recombinant hemagglutinin influenza vaccine provides broader spectrum protection. Expert Rev. Vaccines.

[63] Seong E., Saunders T.L., Stewart C.L., Burmeister M. (2004). To knockout in 129 or in C57BL/6: That is the question. Trends Genet..

[64] Kim E.H., Woodruff M.C., Grigoryan L., Maier B., Lee S.H., Mandal P., Cortese M., Natrajan M.S., Ravindran R., Ma H. (2020). Squalene emulsion-based vaccine adjuvants stimulate CD8 T cell, but not antibody responses, through a RIPK3-dependent pathway. Elife.

[65] Goff P.H., Eggink D., Seibert C.W., Hai R., Martinez-Gil L., Krammer F., Palese P. (2013). Adjuvants and immunization strategies to induce influenza virus hemagglutinin stalk antibodies. PLoS ONE.

[66] Ashkar A.A., Rosenthal K.L. (2002). Toll-like receptor 9, CpG DNA and innate immunity. Curr. Mol. Med..

[67] Tobery T.W., Caulfield M.J. (2004). Identification of T-cell epitopes using ELISpot and peptide pool arrays. Methods Mol. Med..

[68] Knowlden Z.A.G., Richards K.A., Moritzky S.A., Sant A.J. (2019). Peptide Epitope Hot Spots of CD4 T Cell Recognition Within Influenza Hemagglutinin During the Primary Response to Infection. Pathogens.

[69] DiPiazza A., Richards K., Poulton N., Sant A.J. (2017). Avian and Human Seasonal Influenza Hemagglutinin Proteins Elicit CD4 T Cell Responses That Are Comparable in Epitope Abundance and Diversity. Clin. Vaccine Immunol..

[70] Richards K.A., Chaves F.A., Krafcik F.R., Topham D.J., Lazarski C.A., Sant A.J. (2007). Direct ex vivo analyses of HLA-DR1 transgenic mice reveal an exceptionally broad pattern of immunodominance in the primary HLA-DR1-restricted CD4 T-cell response to influenza virus hemagglutinin. J. Virol..

[71] DiPiazza A., Laniewski N., Rattan A., Topham D.J., Miller J., Sant A.J. (2018). CD4 T Cell Epitope Specificity and Cytokine Potential Are Preserved as Cells Transition from the Lung Vasculature to Lung Tissue following Influenza Virus Infection. J. Virol..

[72] Alipour Talesh G., Ebrahimi Z., Badiee A., Mansourian M., Attar H., Arabi L., Jalali S.A., Jaafari M.R. (2016). Poly (I:C)-DOTAP cationic nanoliposome containing multi-epitope HER2-derived peptide promotes vaccine-elicited anti-tumor immunity in a murine model. Immunol. Lett..

[73] Haseda Y., Munakata L., Kimura C., Kinugasa-Katayama Y., Mori Y., Suzuki R., Aoshi T. (2021). Development of combination adjuvant for efficient T cell and antibody response induction against protein antigen. PLoS ONE.

[74] Akkaya M., Akkaya B., Sheehan P.W., Miozzo P., Pena M., Qi C.F., Manzella-Lapeira J., Bolland S., Pierce S.K. (2017). T cell-dependent antigen adjuvanted with DOTAP-CpG-B but not DOTAP-CpG-A induces robust germinal center responses and high affinity antibodies in mice. Eur. J. Immunol..

