# Supplementary material for: R-DOTAP Cationic Lipid Nanoparticles Outperform Squalene-Based Adjuvant Systems in Elicitation of CD4 T Cells after Recombinant Influenza Hemagglutinin Vaccination
Source: Viruses. 2023 Feb 15;15(2):538. doi: 10.3390/v15020538 (PMC9959843; doi:10.3390/v15020538)
Supplement: Supplementary file 1 [file viruses-15-00538-s001.zip › viruses-2158920-supplementary.pdf]

Supplemental Data

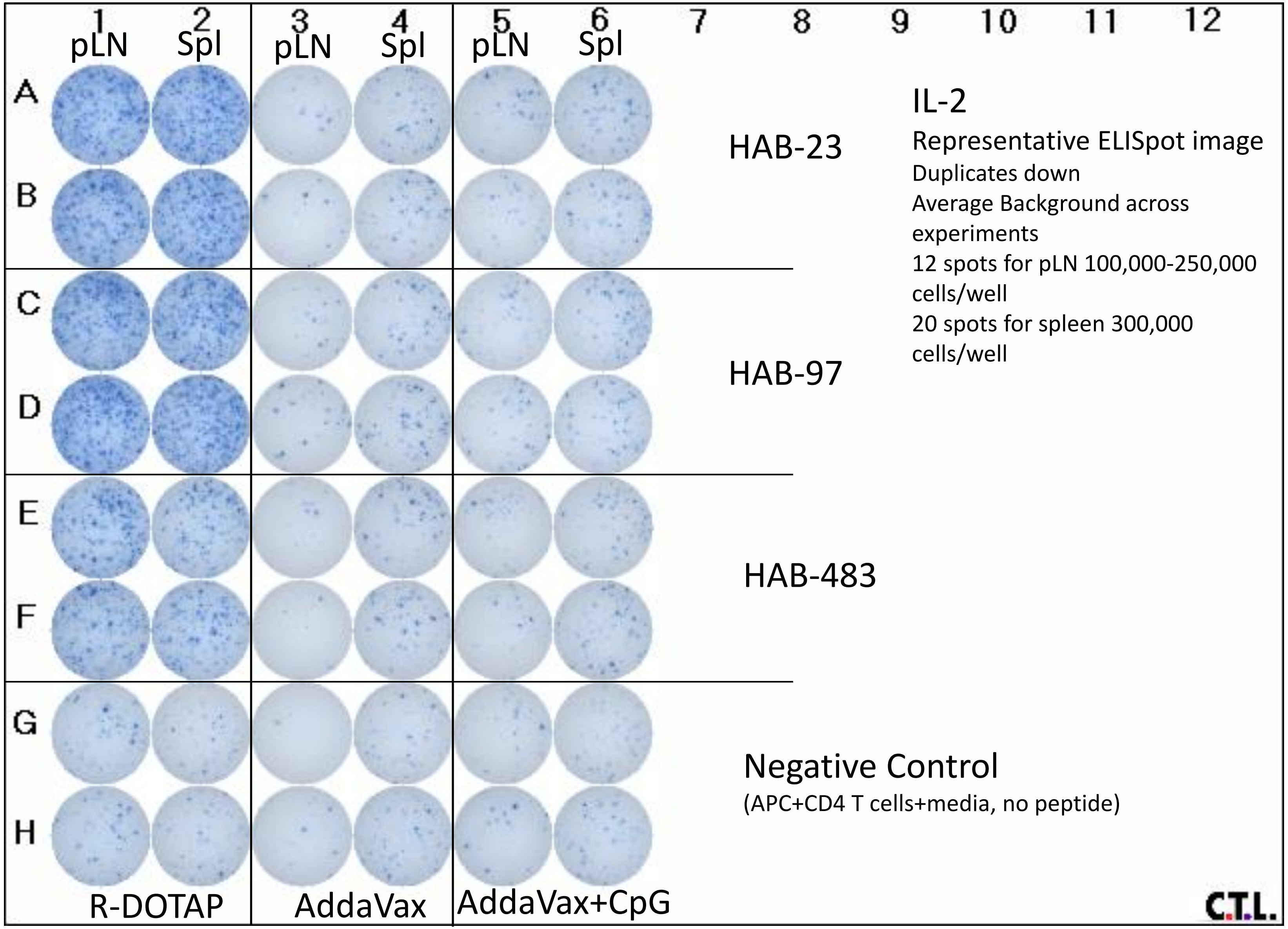

Supplementary Figure 1. Representative plate image for IL-2 ELISpot assay. The plate layout is overlaid on the image with duplicate wells going down the plate, R-DOTAP in columns 1 and 2, AddaVax in columns 3 and 4 and AddaVax+CpG in columns 5 and 6. For each adjuvant CD4 T cells isolated from the draining popliteal lymph node and the spleen were plated side-by-side. The three peptides were plated down the plate as indicated at a final concentration of 5uM. APC and CD4 T cells co-cultured with media and no peptide was used as a negative control for background.

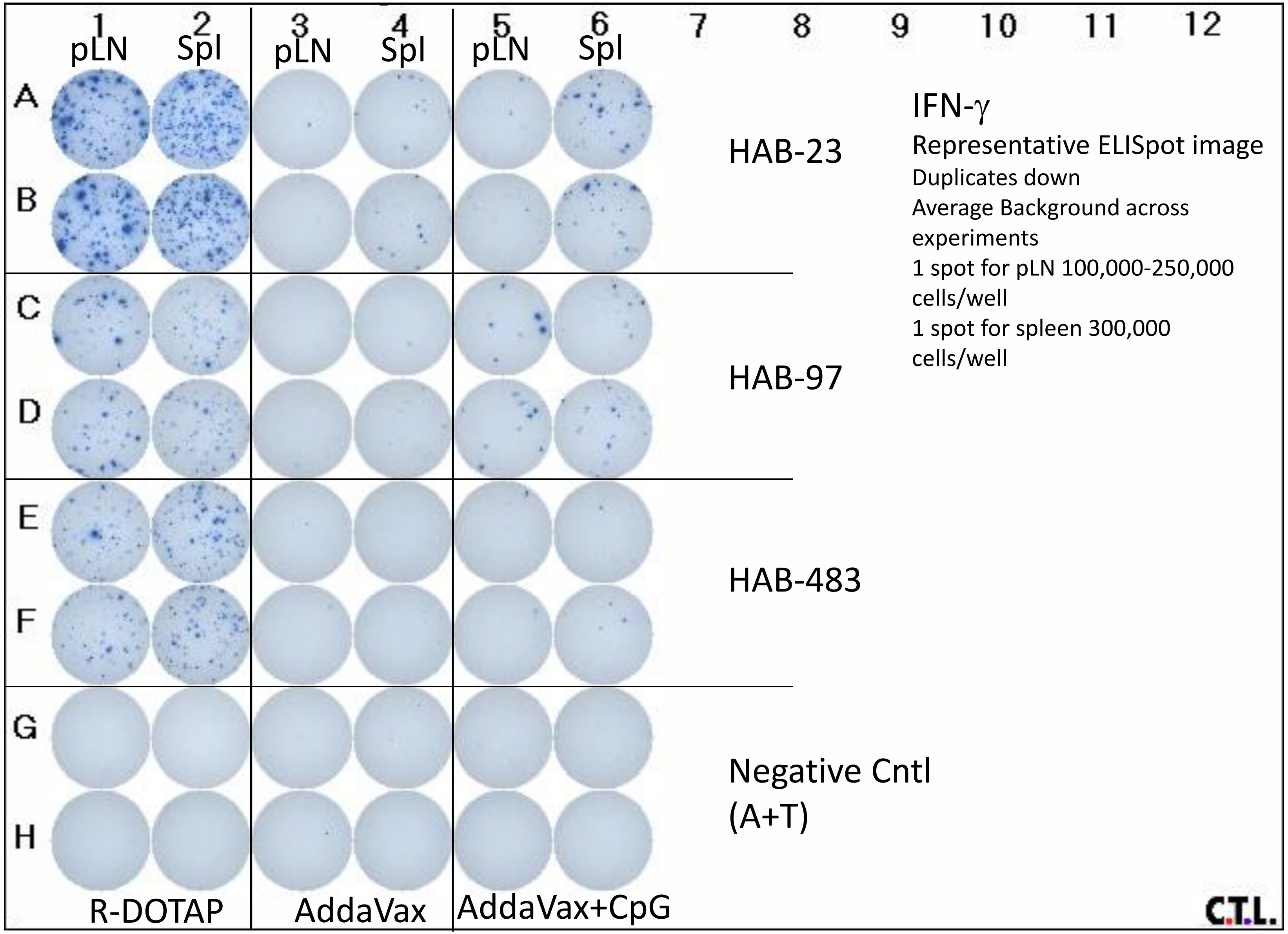

Supplementary Figure 1. Representative plate image for IFN-γ ELISpot assay. The plate layout is overlaid on the image with duplicate wells going down the plate, R-DOTAP in columns 1 and 2, AddaVax in columns 3 and 4 and AddaVax+CpG in columns 5 and 6. For each adjuvant CD4 T cells isolated from the draining popliteal lymph node and the spleen were plated side-by-side. The three peptides were plated down the plate as indicated at a final concentration of 5uM. APC and CD4 T cells co-cultured with media and no peptide was used as a negative control for background.
